# Reduced PHLPP Expression Leads to EGFR-TKI Resistance in Lung Cancer by Activating PI3K-AKT and MAPK-ERK Dual Signaling

**DOI:** 10.3389/fonc.2021.665045

**Published:** 2021-06-08

**Authors:** Wei Wang, Xinhang Xia, Kuifei Chen, Meng Chen, Yinnan Meng, Dongqing Lv, Haihua Yang

**Affiliations:** ^1^ Laboratory of Cellular and Molecular Radiation Oncology, Radiation Oncology Institute of Enze Medical Health Academy, Affiliated Taizhou Hospital of Wenzhou Medical University, Taizhou, China; ^2^ Department of Radiation Oncology, Affiliated Taizhou Hospital of Wenzhou Medical University, Taizhou, China; ^3^ School of Medicine, Shaoxing University, Shaoxing, China; ^4^ Department of Pulmonary Medicine, at Enze Hospital, Affiliated Taizhou Hospital of Wenzhou Medical University, Taizhou, China

**Keywords:** lung cancer, PHLPP, EGFR-TKI resistance, signaling pathway, biomaker

## Abstract

**Background:**

Epidermal growth factor receptor tyrosine kinase inhibitors (EGFR-TKIs) are effective in advanced EGFR-mutation non-small cell lung cancer (NSCLC) but the magnitude of tumor regression varies, and drug resistance is unavoidable. The pleckstrin homology domain leucine-rich repeat protein phosphatase (PHLPP) levels are reduced or lost and acts as a tumor suppressor in many cancers. Here, we hypothesized that PHLPP is a key regulator of EGFR-TKI sensitivity and a potential treatment target for overcoming resistance to EGFR-TKI in lung cancer.

**Methods:**

Cell proliferation and growth inhibition were measured by 3-(4,5-dimethylthiazol-2-yl)-2,5-diphenyltetrazolium bromide (MTT) and colony formation assay. PHLPP- knockdown stable cell lines were generated by lentivirus-mediated delivery of PHLPP shRNAs. The expression of PHLPP mRNA and protein levels was detected by real-time quantitative polymerase chain reaction (qPCR) and Western blotting. Immunohistochemical (IHC) staining was performed to detect the PHLPP expression in clinical patient tissue samples. A transcriptomic assay of genome-wide RNA expressions of PHLPP in NSCLC cell lines according to gefitinib sensitivity was obtained from Gene Expression Omnibus (GEO) database. Murine xenograft model was established to verify the function of PHLPP in gefitinib resistance *in vivo*.

**Results:**

PHLPP highly expressed in gefitinib-sensitive NSCLC cell lines than gefitinib-resistant NSCLC cell lines. In gefitinib-acquired resistance cell line HCC827-GR, PHLPP expression even dramatically reduced. Knockdown of PHLPP in NSCLC cells decreased cell death induced by the EGFR-TKI, while overexpression PHLPP in gefitinib-resistance NSCLC cells can enhance or restore EGFR-TKIs sensitivity. Mechanism study indicated that PHLPP downregulation attenuates the effect of EGFR-TKI on the both AKT and ERK pathway, thereby decreasing the cell death sensitivity to EGFR inhibitors. In xenograft mice, knockdown of PHLPP decreased tumor response to gefitinib and advanced tumor cells re-growth after gefitinib treatment. In clinical, PHLPP expression were reduced in the post-relapse tumor compared to that of pre-treatment, and lower pre-treatment PHLPP levels were significantly correlated with shorter progression-free survival (PFS) in patients with EGFR-mutant lung adenocarcinoma whom treated with EGFR-TKI.

**Conclusions:**

Our data strongly demonstrated that loss of PHLPP function was a key factor of EGFR-TKI resistance in NSCLC. Downregulated PHLPP expression activated PI3K-AKT and MAPK-ERK pathway which strengthened cell survival to EGFR-TKI. Therefore, PHLPP expression level was not only a potential biomarker to predict EGFR-TKIs sensitivity but also as a therapeutic target in EGFR-TKIs therapy, enhancing PHLPP expression may be a valuable strategy for delaying or overcoming EGFR-TKIs drug resistance.

## Background

Lung cancer is the leading cause of cancer-related death worldwide and non-small cell lung cancer (NSCLC) is the most histological subtype of lung carcinoma (1). Lung adenocarcinoma (LUAD) accounts for 60% in lung cancer. Mutations in EGFR are present in 40% to 60% of East Asian-ancestry LUADs (2). EGFR tyrosine kinase inhibitors (TKIs), such as erlotinib, gefitinib, afatinib, osimertinib, and so on, all were widely used in Chinese and other Asian LUAD patients (3–6). Although the achievement of this treatment is significantly effective, the patients’ overall survival after treatment remains poor due to the TKI resistance. Therefore, identifying a reliable biomarker for predicting primary TKI sensitivity/resistance and method to delay acquire resistance onset is essential important.

Many mechanisms of acquired resistance have been identified over the last several decades. One of the common resistance mechanisms is the development of secondary mutation in EGFR, such as T790M mutation, which is detected in more than 50% EGFR-TKIs resistance cases (7). The second category of resistance is associated with re-activation of the two main EGFR downstream signaling pathways PI3K-AKT and MEK/ERK, such as MET was found to promote resistance by reactivating both PI3K-AKT and MEK/ERK signaling despite the inhibition of EGFR (8); In addition, tumor resistance undergoes cell/tumor phenotypic/histological changes, such as epithelial-to-mesenchymal transition (EMT) histological transformation and development toward small-cell lung cancer (SCLC) (9–11). However, resistant mechanism in about 30% recurrence patients is still unknown.

PH domain leucine rich repeat protein phosphatases (PHLPP) comprises of two genes: PHLPP and PHLPP2. Both family members belong to a novel family of Ser/Thr phosphatase, which directly dephosphorylate Akt Ser473 site and inactivate the Akt kinase, and dephosphorylate Raf1 S338 then down regulate the MAPK-ERK signaling (12, 13). PHLPP was downregulated in various tumors, which leads to development of carcinogenesis and cancer progression. In addition, recent studies showed that restoration of PHLPP increased the sensitivity of the colorectal cancer cells to EGFR monoclonal antibodies cetuximab and reverted the resistance (14). In chemotherapy, downregulation of PHLPP contributed to chemosensitivity, achieved cancer cells sensitive to oxaliplatin and paclitaxel resistance in colon cancer cell (15). In our own previous clinical results, we found that high PHLPP expression predicted longer duration of acquired resistance in lung adenocarcinoma patients with EGFR tyrosine kinase inhibitors (16). However, the role of PHLPP in resistance to EGFR-TKI in EGFR-mutation lung cancer remains undefined. Given the aforementioned studies demonstrating that PHLPP plays an important role in tumor progression and treatment resistance, it seems reasonable to speculate that TKI could not fully inhibit PHLPP loss-mediated AKT S473 phosphorylated to cause TKI resistance in PHLPP downregulation EGFR-mutant lung cancer cells.

Since the critical role of PHLPP function dampens both PI3K-AKT and MAPK-ERK proliferation signaling pathways, several resistance mechanisms toward EGFR-TKI are through reactivation of PI3K-AKT and MAPK-ERK. We hypothesized that PHLPP might have an important role involved in the underlying mechanism, changing the EGFR-TKI sensitivity in harbored EGFR mutation NSCLC. In the present study, we will determine the role of PHLPP in EGFR TKI resistance lung cancer after EGFR-TKI therapy *in vitro* and *in vivo*.

## Materials and Methods

### Cell Culture and Reagents

H1650, H1975, and HCC827 cell lines were purchased from the American Type Culture Collection (ATCC). All cell lines were test for mycoplasma and were negative for mycoplasma using mycoplasma Plus PCR Primer Set. All cells were cultured in a 37°C, 5% CO_2_-humidified incubator using RPMI 1640 medium with 10% fetal bovine serum (FBS; Gibco) and penicillin (100 U/ml)/streptomycin (100 μg/ml) while HERK-293T were maintained in DMEM-completed medium. Gefitinib, erlotinib, afatinib, and osimertinib, purchased from Selleck Chemicals, were dissolved in dimethylsulfoxide (DMSO) to a final concentration of 10 mmol/L and stored at −20°C.

### Generation of Gefitinib-Resistant HCC827 Cells *In Vitro*

To generate gefitinib-resistant cell line, HCC827 cells were generated and exposed to increasing concentrations of gefitinib, similar to previously described methods (17). Gefitinib concentrations were increased stepwise from 10 nM to 2 μM when the cells resumed growth kinetics similar to the untreated parental cells. Fresh drug was added every 72 h. Cells that are able to grow in 2-μM gefitinib were obtained 6 months after initial drug treatment. To confirm the emergence of a resistance, MTT assay was performed following growth at each concentration after allowing the cells to grow in a drug-free condition for at least 3 days. Control HCC827 cells were maintained concomitantly without gefitinib, and their sensitivity to gefitinib was examined every month.

### Cell Cytotoxicity Assay

Following the indicated drug treatment, cell viability was assessed by MTT assay. Cell suspensions (2–5 × 10^3^/100 μl/well) were seeded into 96-well cell culture plate overnight. The following day, various concentrations of indicated drugs were added. After incubation for 72 h, cells were fixed with 4% formaldehyde for 20 min, stained with 0.5% crystal violet solution containing 20% (vol/vol) methanol in PBS, then the plates were washed with deionized water and dried completely. Crystal violet absorbed by the cells solubilized in 10% (vol/vol) acetic acid were add into each well and the absorbed crystal violet optical density was detected at 595 nm by a microplate reader. Triplicate wells were tested at each drug concentration.

The 50% inhibitory concentration (IC50) value was calculated using a non-linear regression model with a sigmoidal dose response using GraphPad Prism 8.0 for Windows (Prism GraphPad software, La Jolla, CA, USA). The IC50 value, defined as the concentration giving a 50% reduction in absorbance, was calculated from the survival curves. The data were graphically displayed using GraphPad Prism version 8 for Windows (Prism GraphPad software, La Jolla, CA, USA). The curves were fitted using a non-linear regression model with a sigmoidal dose response.

For colony formation, 5 × 10^4^ cells/well were seeded in six-well plate, exposed to indicated EGFT-TKI treatment and maintained in RPMI-1640 medium supplemented with 10% FBS for 14 days. Plates were fixed with 4% formaldehyde for 20 min at room temperature and stained in 0.5% crystal violet for 2 h.

### Generation of Genetically Modified Cell Lines

The lentivirus-mediated delivery of PHLPP shRNAs selection for stable PHLPP knockdown cell line was performed as described previous (15). pLKO-sh-PHLPP (sh97) and control vector were gifted from Dr. Gao Lab.

Lentiviral particles were produced by co-transfection of either pLKO-sh-PHLPP (sh97) or shControl Vector with pMD2G (Addgene #12259) and psPAX2 (Addgene #12260) into 293T cells using PEI (1 µg/µl), viral supernatants were harvested 48 and 72 h (second collection) after transfection, filtered with a 0.45-μm cellulose acetate filter (Millipore Sigma, Cat#SE1M002M00). Viral transductions were performed for 16 to 24 h in the presence of 8 µg/ml polybrene. Stable knockdown cells were selected 48 h after transduction with 2 μg/ml puromycin for 10 to 14 days and confirmed by qRT-PCR and Western blot.

For stable PHLPP overexpression cell line, the retroviruses-mediated delivery of overexpression plasmid encoding HA-tagged PHLPP (pBabe-puro-HA-PHLPPβ, From Dr. Gao, University of Kentucky) were transfected and selected with puromycin. Non-targeted retroviral vector was used as control (pBabe-puro-vector).

### Apoptosis Assay

Either vehicle (DMSO) or EGFR-TKI treated cells were resuspended using phosphate-buffered saline (PBS) and stained with AnnexinV–FITC and propidium iodide (PI) using the BD apoptosis detection Kit (BD Biosciences, #556547) according to manufacturer’s instructions. Data were acquired using a CytoFlex S Flow Cytometer Beckman Coulter and analyzed with FlowJo software (TreeStar Inc.)

### Western Blotting

The cells were collected and lysed in lysis buffer (50 mM Tris-HCl, pH7.4; 100 mM NaCl; 1 mM EDTA; 1 mM EGTA; 1 mM NaF; 0.1% SDS; 0.5% sodium deoxycholate; 1% Triton-X-100; 10% glycerol; protease and phosphatase inhibitor cocktails) on ice. Protein quantification was conducted with the BCA assay kit (Bio-Rad, Hercules, CA, USA). Equal amount of total protein lysates (about 25 µg) was subjected to 8% to 15% SDS-PAGE and transferred onto a polyvinylidene fluoride (PVDF) membrane. The PVDF membrane then was blocked with 5% non-fat milk for 1 h followed by incubation with the specific primary antibodies at 4°C overnight. The primary antibodies are the following: PHLPP (1:2000, ProteinTech 22789-1-AP), PHLPP2(1:2000, ProteinTech25244-1-AP), phospho-EGFR Y1068 (1:1000, CST#3777), EGFR (1:1000, CST#4627), phosphoERK-p44/42(1:2000, CST#4370), ERK1/2(1:1000, CST#4695) phospho-AKT S473 (1:2000, CST #4060), AKT(CST#4685), β-Actin (CST#8457). The membrane was then incubated with the horseradish peroxidase-labeled secondary antibody (ab205718, Abcam) for 1 h at room temperature. Signal was detected using the ECL detection kit (Thermo Fisher) and a FluoChem digital imaging system (Alpha Innotech, San Leandro, CA, USA). Band intensity was quantified by the software Image J (NIH, Bethesda, MD, USA).

### Quantitative Real-Time PCR (qRT-PCR)

RNA was extracted by column purification kit (Qiagen RNeasy), and then retro-transcribed into first-strand cDNA using the High Capacity cDNA Reverse Transcription Kit following the manufacturer’s protocol (Thermo Fisher Scientific, CAT#4368814). qRT-PCR was performed using SYBR™ Select Master Mix (LiCfe Technologies, cat# 4472908) on an ABI 7500 Fast Real-Time PCR platform (Applied Biosystems). Relative mRNA levels of each gene showed were normalized to the expression of the housekeeping gene GAPDH. Real-time PCR Primer as follows: PHLPP (Forward, CCTCATCCGCTTCTATGCAGG; Reverse, GCATCTTGCCTTTACGGAC); PHLPP2 (Forward, ATGGAGCAGACACTACCACTG; Reverse, GCAAAGGACGAGATGTAAGTCA); GAPDH (GGAGCGAGATCCCTCCAAAAT; Reverse, GGCTGTTGTCATACTTCTCATGG). Each sample was amplified in triplicate, and data were analyzed by relative quantitation using the ΔΔCt method and normalization to GAPDH.

### Bioinformatics Analysis of GSE4342 Database

PHLPP and PHLPP2 transcription expression of 29 NSCLC cell lines in this study were obtained from the publicly available data set GSE4342 (18).

### Xenograft Mouse Studies

All mouse studies were conducted in accordance with the standards of the Institutional Animal Care and Use Committee under a protocol approved by the Institutional Animal Care and Use Committee. Either 5×10^6^ shRNA-Control HCC827 cells or PHLPP shRNA knockdown HCC827 cells were inoculated into the low-left quadrant of the flank of 6- to 8-week-old female nude mice. Mice were monitored daily general condition and body weight. Tumor sizes were measured every other day using calipers. Volume was calculated using the formula L × W^2^ × 0.52, where L is the longest dimension and W is the perpendicular dimension. Mice were randomized to receive gefitinib or vehicle treatment once the mean volume was reached approximately 300 to 400 mm^3^. Gefitinib were administered at doses/schedules (equal to or below the maximum tolerated doses) previously reported to be effective in tumor growth inhibition (19). The experiment was terminated when the tumor in the either treatment group began to progress or when the tumor mean volume of vehicle control group reached 2000 mm^3^.

### Biopsies of Human NSCLC and Immunohistochemistry (IHC)

This study was approved by the institutional research ethics board. All patients provided their written informed consent for permitting analyses of tissues. Patients with advanced EGFR mutation NSCLC who received EGFR-TKI as first-line treatment were identified from our institution, following clinical practice, radiographic progression on EGFR-TKI therapy underwent re-biopsy, availability of paired pre- and post-TKI FFPE biopsies. IHC was performed as described previously (20). Briefly, following deparaffinization and rehydration of the slides, antigen retrieval was performed using 10 mM sodium citrate (pH 6.0) for 5 min. After endogenous peroxidase activity was blocked with 3% hydrogen peroxide, samples were incubated with PHLPP (1:100, ProteinTech, 25244-1-AP) overnight at 4°C, HRP polymer for detection was subsequently applied onto the slides. Sections were then incubated with DAB refine for visualization and counterstained with hematoxylin. As previously defined, the IHC score of tumor were according to the intensity of staining with Fourier system (level 0–3: negative = 0, weakest = 1, moderate = 2, strong = 3) [14].

### Statistical Analysis

For the *in vitro* studies, statistical analysis was performed using Student’s *t* test (two-tailed) or one-way ANOVA. P ≤ 0.05 was considered statistically significant. Values were expressed as mean ± standard errors of the means (SEMs).

## Results

### PHLPP Expression Level Positively Correlated With Sensitivity to EGFR-TKI

**Figure 1:**
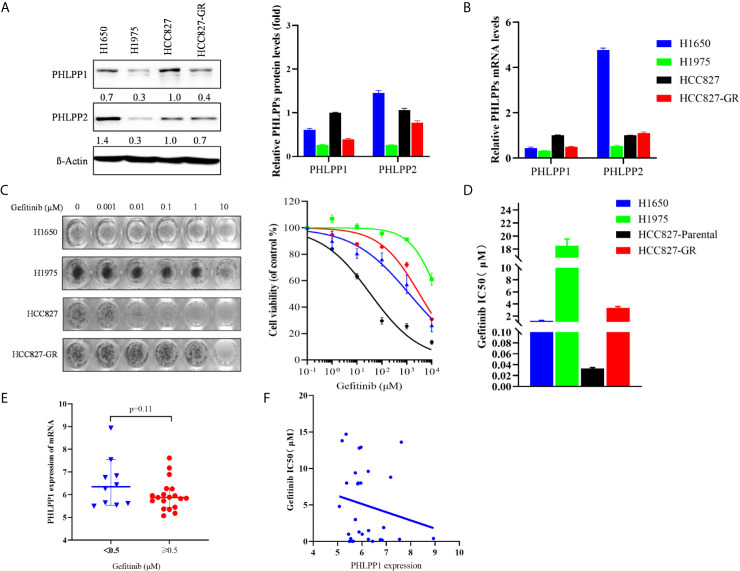
PHLPP expression positively correlated with sensitivity to EGFR-TKI. **(A)** Immunoblot of PHLPP and PHLPP2 in NSCLC cell lines. β-actin was used as a loading control. The relative intensity of PHLPP levels is determined and relative to HCC827 cells. Data are from 3 independent experiments and are actin normalized and expressed relative to HCC827 cells as the mean ± SEM. **(B)** Quantitative RT-PCR analysis of PHLPPs mRNA levels in NSCLC cell lines and expressed relative to HCC827 cells as the mean ± SEM. **(C)** Representative image for proliferation inhibition by MTT assay(left). Five NSCLC cell lines treated with the increased concentrations of gefitinib (0-10 μM), cell viability relative to vehicle treated after 72 h. Each data point represents the mean ± SEM of at least 3 wells(right). **(D)** The mean IC50 value of gefitinib from five NSCLC cell lines as measured by growth inhibition assays. Data are from three independent experiments as mean ± SEM. **(E)** Box plots depicting PHLPP RNA expression in 29 NSCLC cell line form GSE4342 using the 0.5 μM gefitinib IC50 values as cut-off value. **(F)** Dot plot depicting the correlation of gefitinib IC50 value and the expression level of PHLPP in 29 NSCLC cell lines from GSE4342.

We determined that PHLPP expression level correlated with EGFR-TKI sensitivity in NSCLC cells. Western blotting showed that PHLPP expression level in the EGFR-TKI sensitivity cell line HCC827 was the highest among H1650, H1975, and HCC827 gefitinib-resistant (HCC827-GR) cells (**Figures 1A, B**), but cannot detect differences of the PHLPP2 protein levels in these cell lines. A similar result was determined about the PHLPP mRNA expression levels in these four cell lines (**Figure 1C**). The MTT assay was used to determine the inhibitive concentration of 50% cell viability (IC50) for gefitinib, as shown in **Figures 1C, D**. The HCC827 cells exhibited the lowest IC50 value for gefitinib (0.035 μM) compared with the IC50 value for gefitinib in the H1650, H1975, and HCC827-GR were 1.27, 18.51, and 3.22 μM, respectively.

Next, we analyzed the gene expression of PHLPP in NSCLC cell line according to gefitinib sensitivity from the database of GSE4342 (18), PHLPP mRNA expression was higher in the gefitinib-sensitive group (IC50 <0.5μM) compared to the gefitinib-resistant group (IC50≥0.5μM), the mean value of mRNA expressions were 6.5± 0.35 and 5.9 ± 0.15 (**Figure 1E**). PHLPP mRNA expression was negatively correlated with gefitinib IC50 value, cell lines with high expression of PHLPP were found to be less IC50 value of gefitinib, with a Pearson correlation score of −1.123 (**Figure 1F**). These findings suggested that PHLPP might be related to EGFR-TKI sensitivity in NSCLC cells.

### PHLPP Downregulation Re-activates ERK1/2 and AKT Signaling

**Figure 2:**
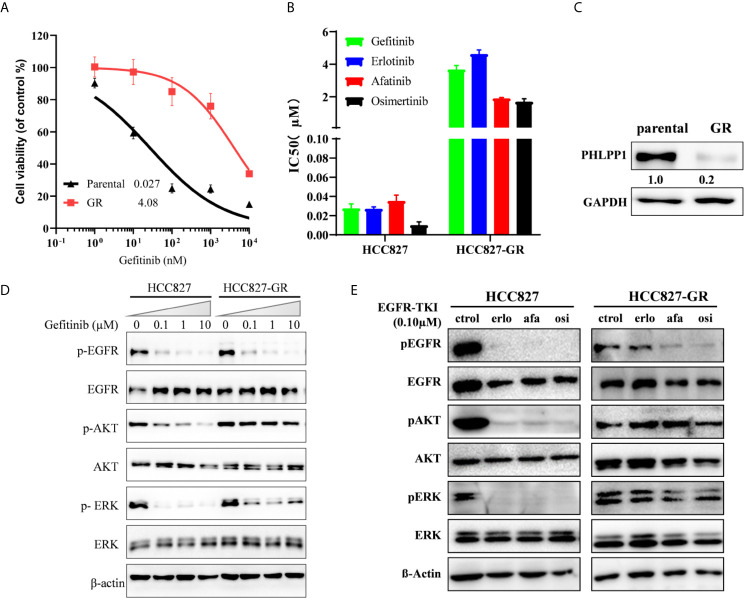
Acquired loss of PHLPP expression in EGFR mutation cell line resistant to gefitinib. **(A)** Cell growth-inhibition assays demonstrate the resistance of HCC827 to gefitinib (HCC827-GR), relative to parental control HCC827. Parental HCC827 and HCC827-GR cells were subjected to a 72-h growth inhibition assay in increasing concentrations of gefitinib. Results are presented as percentage of survival compared with cell grown in the vehicle treated cells. **(B)** HCC827-GR also confers cross-resistance to another EGFR-TKI. The mean IC50 values of four EGFR-TKI for 72 h growth inhibition assay in HCC827 and HCC827-GR cells. Data are representative of three independent experiments. **(C)** PHLPP Protein expression in HCC827 and HCC827-GR cell lines were detected by western blotting. **(D)** HCC827 and HCC827-GR cell lines were treated with increase concentration gefitinib for 24 h. The cells were lysed and indicated protein were detected by western blotting. **(E)** HCC827 and HCC827-GR cell lines were treated with indicated EGFR-TKI for 24 h. The cells were lysed and indicated protein were detected by western blotting.

We generated a gefitinib-resistant (GR) of the EGFR-mutant HCC827 (Del E746_A750) cell line using previously established methods. Several HCC827 gefitinib-resistant (GR) clones were established and confirmed to be gefitinib-resistant (**Figure 2A**), the IC50 of gefitinib in HCC827-GR increased ~100 fold compared to HCC827-parental. The HCC827-GR cells are also cross-resistant to other EGF receptor kinase inhibitors like erlotinib, afatinib, and osimertinib (**Figure 2B**). All HCC827-GR cells still harbored the EGFR del E746_A750 without T790M mutation. A significantly decreased expression of PHLPP in HCC827-GR cells compared with parental cells is shown in **Figure 2C**, but no difference was observed in PHLPP2 between these two cell types (data not shown).

To investigate the underlying mechanism how HCC827-GR is resistant to the EGFR TKI, lysates from both parental and GR cells treated with gefitinib were blotted with antibodies. We found that gefitinib still inhibited EGFR phosphorylation in the HCC827-GR cells, as well as HCC827 parental, the inhibition was decoupled from inhibition of downstream signaling both AKT and ERK phosphorylation (**Figure 2D**). Similar to gefitinib, another EGF receptor kinase inhibitor, erlotinib, afatinib, and osimertinib, was only able to inhibit EGFR phosphorylation but not ERK1/2 and AKT phosphorylation in the HCC827-GR cells (**Figure 2E**). These findings suggest that gefitinib-related EGFR TKI resistance in HCC827 cells adopted a new mechanism by reactivating PI3K-AKT and MAPK/ERK signaling, which may cause adaptive downregulation of PHLPP with long-term exposure to gefitinib.

### PHLPP Loss Leads to Increased EGFR-TKI Resistance *In Vitro*

**Figure 3:**
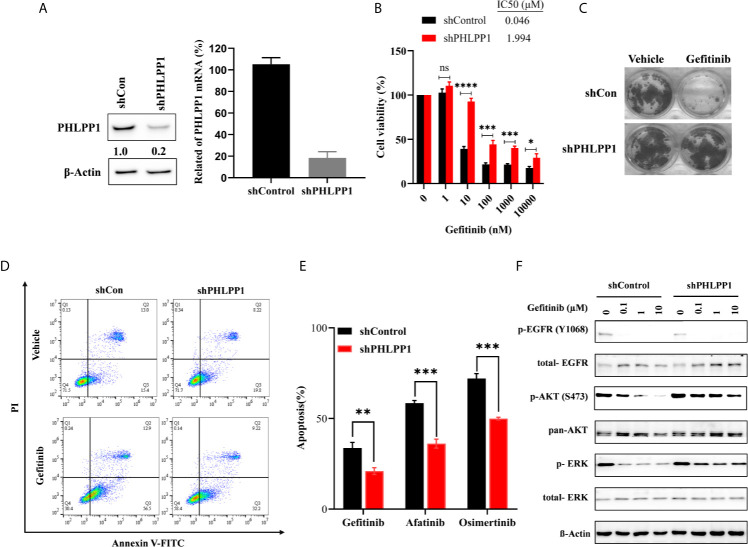
Knockdown .of PHLPP decrease sensitive to EGFR-TKI in EGFR sensitive cell line. **(A)** Knockdown efficiency of PHLPP in HCC827 cell line as determine by both at mRNA level and protein level using quantitative RT-PCR and immunoblotting, respectively **(B)** IC50 values (μM) were based on data obtained from growth inhibition assay. **(C)** Longer-term colony formation assay using to determine downregulation PHLPP expression enhances cell survival **(D)** Detection of apoptosis with annexin V/flow cytometry. **(E)** HCC827-shControl and HCC827-shPHLPP cell lines were exposed to indicated EGFR-TKIs for 24 h then harvested for detection of apoptosis with annexin V/flow cytometry. The apoptosis of % are mean ± SE of triplicate assay. **(F)** HCC827-shControl and HCC827-shPHLPP cell lines were treated with increase concentration gefitinib for 24 h. The cells were lysed and indicated protein were detected by western blotting.

Given that PHLPP is downregulated in gefitinib-resistant cell line, we next to validated the possible role of PHLPP in gefitinib resistance. Stable PHLPP knockdown HCC827 cells were generated using lentiviral-based shRNA. PHLPP rival particle transductions did efficiently suppress PHLPP protein and mRNA expression levels (**Figure 3A**). MTT assay was used to determine the inhibition of cell proliferation in the short term induced by gefitinib in these PHLPP knockdown cells. The IC50 value was 43-fold, which increased after gefitinib treatment. To test longer-term gefitinib effects in PHLPP silent HCC827 cells, we performed colony formation assays, in which PHLPP knockdown cells were culture in the presence of gefitinib for 14 days. PHLPP knockdown substantially enhanced survival in the presence of gefitinib compared to PHLPP shRNA control cell lines (**Figure 3B**). In addition, we found gefitinib-induced apoptosis was significantly decreased in PHLPP knockdown cells (**Figure 3D**). Another EGFR-TKI-induced apoptosis was decreased similar to gefitinib (**Figure 3E**). To explore the mechanism of gefitinib resistance induced by PHLPP loss, we next examined blocked phosphorylation level of EGFR, AKT, and ERK1/2 induced by gefitinib in both PHLPP knockdown and control HCC827 cells. We found that EGFR phosphorylation was completely abolished in both control and PHLPP-knockdown cells, but AKT and ERK1/2 phosphorylation substantially retained in the presence of gefitinib in PHLPP knockdown cells (**Figure 3F**). Taken together, our finding revealed that PHLPP downregulation attenuated the effect of EGFR-TKI, decreased the dephosphorylation levels of both AKT and MAPK, reactivated AKT and MAPK pathway *in vitro*, therefore, PHLPP downregulation decreased cell sensitivity to EGFR-TKI.

### Overexpression of PHLPP Increase Sensitivity to EGFR-TKI

**Figure 4:**
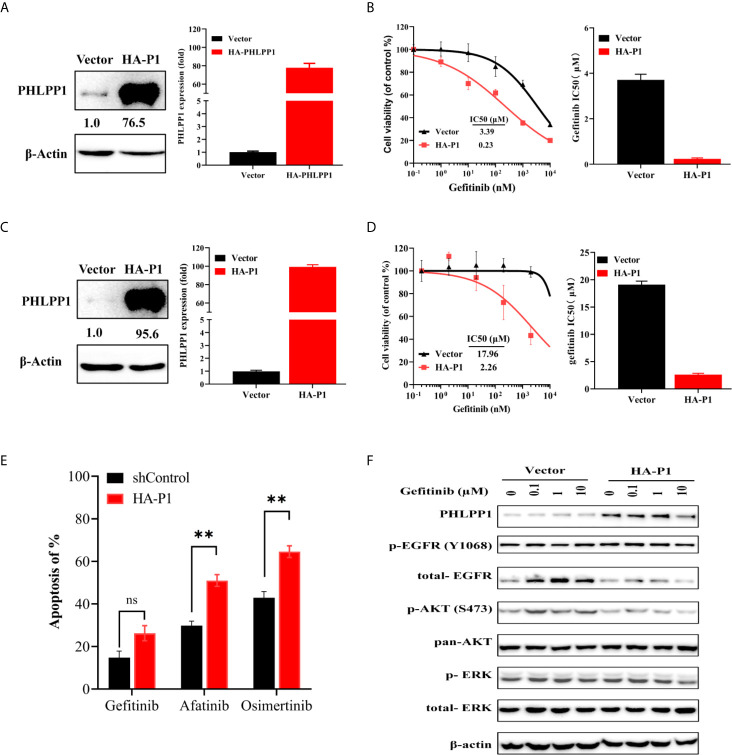
Overexpression of PHLPP increase sensitivity to EGFR-TKI in gefitinib resistance cell line. **(A)** Overexpression efficiency of PHLPP in HCC82-GR cell line as determine by both at mRNA level and protein level using quantitative RT-PCR and immunoblotting, respectively **(B)** Cell-growth inhibition assay. All experiments were repeated at least three times. **(C)** Overexpression efficiency of PHLPP in H1975 as determine at protein level using immunoblotting (left) and fold changes from triplicate experiments (right) **(D)** Cell-growth inhibition assay. The gefitinib IC50 value are mean ± SE of triplicate assay (right). **(E)** HCC827-GR cell were exposed to indicated EGFR-TKIs for 24 h then harvested for detection of apoptosis with annexin V/flow cytometry. The apoptosis of % are mean ± SE of triplicate assay statistical testing was performed by two-sided t-test; **P < 0.005, **(F)** H1975-Vector and H1975-HA-PHLPP cell lines were treated with increase concentration gefitinib for 24 h. The cells were lysed and indicated protein were detected by Western blotting.

We next evaluated whether overexpression of PHLPP would restore sensitivity to EGFR-TKIs in the gefitinib resistance HCC827-GR cells. We established stable PHLPP overexpression cell line by using retrovirus-based transduction in HCC827-GR cells (**Figure 4A**). PHLPP was increased by about 77-fold after transduction. Overexpression of PHLPP was able to restore HCC827-GR sensitivity to gefitinib with a 17-fold decrease in the gefitinib IC50 compared to HCC827-control cells (**Figure 4B**). Similar results were obtained in a gefitinib intrinsic resistance cell line H1975 (**Figure 4C**), the IC50 value of gefitinib decreased by 8-fold in PHLPP overexpression cells (**Figure 4D**). In addition, overexpression of PHLPP in H1975 significantly increased other EGFR receptor kinase inhibitors, afatinib and osimertinib-induced apoptosis (**Figure 4E**). Although EGFR phosphorylation was not inhibited by gefitinib, both AKT and ERK1/2 phosphorylation were effectively inhibited in PHLPP overexpression of H1975 cells (**Figure 4F**). Collectively, these findings strongly revealed that the ability of PHLPP to forestall and restore the onset of resistance to EGFR inhibitor *via* inhibiting EGFR downstream activation in both PI3K-AKT and MAPK-ERK signaling pathways.

### PHLPP Inhibition Advanced the Onset of Resistance to Gefitinib *In Vivo*

**Figure 5:**
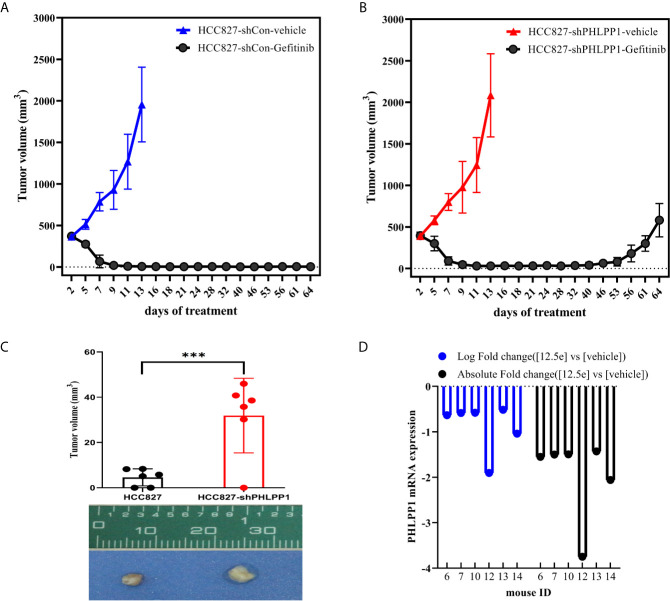
Effect of PHLPP suppression on EGFR-TKI sensitivity *in vivo*. **(A, B)**. Xenografts in *nu*/*nu* mice were generated from either HCC827-shControl **(A)** or HCC827-shPHLPP cells **(B)**. Gefitinib (150mg/kg) was administered by oral gavage, and tumors volume were measured every other day. Mean tumor volumes were shown. **(C)** The mean residue tumor volume after 21-day course gefitinib treatment statistical testing was performed by two-sided t-test; ***P < 0.0005. **(D)** The log fold and absolute fold change of PHLPP mRNA from a previously study have been published from Zhang et al.

To determine whether PHLPP affect cell sensitivity to gefitinib *in vivo*, we conducted tumor xenograft model using subcutaneously tumor planting and following a 21-day gefitinib treatment (150 mg/kg, daily). The tumor growth rates were similar in both PHLPP knockdown and control HCC827 cells derived tumors (**Figures 5A, B**). Over 21-day gefitinib treatment, we found three of six mice in the control group but only one of six mice in PHLPP knockdown group had complete tumor regression (as detected by palpation), the mean tumor volumes in PHLPP knockdown group were higher than in control group (4.6 mm^3^ *vs.* 31.9 mm^3^, **Figure 5C**). Moreover, after the stop of gefitinib treatment, we observed no tumor growth up to 40 days in control group (**Figure 5A**) but tumor grow again within 1 month in PHLPP knockdown group (**Figure 5B**). Furthermore, we analyzed a microarray expression profiling of previously study that have been published by Zhang et al. (21), in which 17 xenograft tumors treated with erlotinib as well as two vehicle-treated control tumors have been conducted. We found that there were 6 of the 17 (35%) tumors with downregulation in HCC827 erlotinib-resistant tumor (**Figure 5D**). On the other side, PHLPP upregulation was not observed in those erlotinib-resistant xenograft mice (21).

Taken together, these results further validation of reduced PHLPP expression alter tumor response to treatment and predictive onset of tumor recurrence *in vivo*.

### PHLPP Loss Is Associated With Resistance to EGFR Inhibitor and Poor Outcome in EGFR Mutant Patients

**Figure 6:**
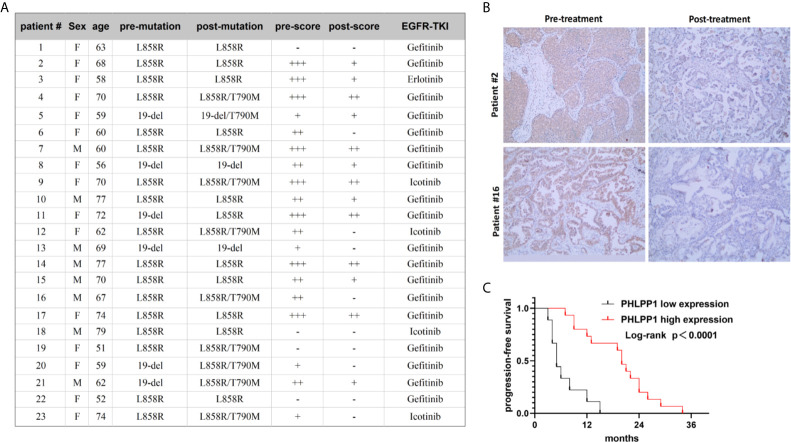
Reduced PHLPP expression contributed to gefitinib resistance in clinical patients’ tissue. **(A)** Clinical characteristics of 23 paired samples from patients with EGFR mutation lung cancer before and after the resistance to EGFR inhibitors. **(B)** Representative IHC Images of human EGFR mutation NSCLC tumor tissues with different PHLPP alteration stained with PHLPP antibodies. **(C)** Progression-free survival of patients receiving first line EGFR-TKI with high or low PHLPP expression level (as assessed by immunohistochemical analysis). Statistical significance was determined by Log-rank test.

To assess whether PHLPP expression associate with resistance to EGFR-TKI in clinical, we detected PHLPP expression levels in tumor samples using IHC technology, these pre- and post-development samples were from patients who carry EGFR common mutations. Matched number of normal tissues as control. In 23 pretreatment and post-relapse tumor sections, we detected 10 of 23 (43.5%) patients with T790M mutation (**Figure 6A**), 18 of 23 (78.3%) patients showed decreased PHLPP expression at the time of the radiographical progression compared to pre-treatment (**Figure 6B**), none of patients with increased PHLPP expression. Of these 18 decreased PHLPP patients, 8 (44.4%) patients with a T790M second mutation, which was similar as observed from those patients without a T790M second mutation. None of these were included in our previously published cohort of EGFR-TKI treatment patients (16).

In addition, to evaluate whether PHLPP is involved in the primary resistance in patients as well, we tested the PHLPP expression level taken at diagnosis using IHC. Using the median as cutoff, we found that PFS was drastically shorter in low PHLPP expression group compared to high expression group (median PFS of 5.0 months *vs* 20.0 months, p =0.0001, **Figure 6C**). Taken together, these clinical data suggest that PHLPP expression level significantly decreased after EGFR-TKI therapy in clinical, it also can be used as a biomarker to predict the patient response to EGFR-TKI.

## Discussion

EGFR-TKI has demonstrated remarkable clinical activity in EGFR mutation NSCLC and has transformed the treatment paradigm for this highly prevalent disease in Asian populations. Despite this impact, intrinsic and acquired resistance to EGFR inhibitors remains a major challenge in the clinic. In the present study, we have identified loss of the PHLPP tumor suppressors as mechanisms of resistance to EGFR-TKI in NSCLC. Furthermore, PHLPP levels may influence the initial response to TKIs, as low as PHLPP in pre-treatment specimens is strongly associated with reduced PFS for patients with EGFR-mutant lung adenocarcinomas treated with EGFR TKI.

A major clinical challenge in targeting EGFR-mutation NSCLC is resistance to EGFR blockade by multiple molecular mechanisms. The most frequents cause of EGFR-TKI resistance is the secondary mutations in EGFR, such as T790M attributed to the first- and secondary-generation EGFR-TKIs (17), C797S mutation confers resistance to irreversible third-generation EGFR-TKI (22). EGFR-independent mechanisms include bypass pathway or downstream activation by amplification of MET (8, 23), CRKL (24), HER2 (25), SMO (26), ERK (27) and KRAS (28)et al. Other altered molecular mechanisms involved in EGFR blockade resistance are transformation to small-cell lung cancer, epithelial-mesenchymal transition (10, 29–31), and the activation of immune system and other immunosuppressive factors, such as PD-L1 (32). In addition, a recently study reported stress hormone activation of β2-AR promotes EGFR TKI resistance in cell lines and in mouse models of EGFR mutant NSCLC and that this can be blocked by anti-IL-6 antibodies or β-blockers (33).

PHLPP downregulation occurs in several type cancers and is associated with poor clinical outcome and treatment resistance. In the present study, we found that PHLPP expression level positively correlated with EGFR-TKI sensitivity in EGFR mutation lung cancer. Among three EGFR mutation cell lines, we found that the expression of PHLPP in HCC827 cell line was the highest, H1975 cell line was the lowest, whereas gefitinib IC50 in H1975 cell line was highest and HCC827 cell line was lowest, respectively. Overexpression of PHLPP protein improves the efficacy of PI3K inhibitor (34)and rapamycin in colon cancer cells (35). Another study demonstrated that the level of PHLPP expression regulates drug sensitivity in colon cancer cells and that downregulation of PHLPP contributes to hypoxia-induced chemoresistance (15). More recently, it has been shown that PHLPP expression decreased in proteasome inhibitor bortezomib-resistance cells and bone marrow sample of patients with multiple myeloma, and overexpression of PHLPP partially increased bortezomib sensitivity in multiple myeloma cell lines (36). Here, we showed that overexpression of PHLPP in EGFR-TKIs resistance cell lines increased apoptosis induced by EGFR inhibitors. Downregulation of PI3K-AKT signaling is necessary for EGFR-TKI to effectively induce apoptosis (16). PHLPP is discovered for its tumor suppressor function in dampening both PI3K-AKT survival and ERK proliferative pathways (12, 13). It has been shown that AKT or/and ERK reactivation *via* varied mechanisms played important role in EGFR-TKI resistance (37). However, the mechanisms of reactivation of PI3K-AKT and MAPK-ERK pathway are unclear. Consistent with previous studies, we found that PHLPP is downregulated in gefitinib resistance cell lines accompanied by reactivation of AKT and ERK. Overexpression of PHLPP sensitize EGFR-TKI resistant cell line to EGFR inhibitors. Conversely, downregulation of PHLPP in EGFR-TKI sensitive cell lines decrease EGFR inhibitors induced apoptosis.

Epigenetic dysregulation is in partly attributed to cancer treatment resistance, epigenetic drugs combined with molecularly targeted therapy could have important roles in reversing acquired therapy resistance. It has been shown that combinations of HDAC inhibitor such as vorinostat, panobinostat, and EGFR-TKIs to overcome EGFR-TKI resistance is associated with the BIM polymorphism in EGFR-mutant NSCLC (38, 39). Another study has shown that osimertinib in combination with BET inhibitors JQ1 enhance the anti-tumor response in HER2 aberrations NSCLC (40). A preclinical research found that combination treatment with the EHMT2 inhibitor and erlotinib resulted in enhanced antitumor effects *via* reverse the silencing of PTEN and activation of the AKT pathway in EGFR‐TKI‐resistant cells in a preclinical EGFR-TKI-resistance model (41). One limitation of our study is that we did not explore epigenetic. How chronic exposure to EGFR-TKI induces PHLPP expression decrease remains unclear and potentially involves transcription factors or chromatin modifying enzymes that regulate EGFR-TKI response. Dong et al. showed blockade of binding the transcription factor Sp1 to the PHLPP promoter contributes to DNA methylation-mediated suppression of PHLPP and promotes Akt activation in melanoma (42).

## Conclusions

In conclusion, our data point to activation of PI3K-AKT and MAPK-ERK signaling pathway by PHLPP loss as a potentially common mechanism of resistance to EGFR-TKI, nominating PHLPP as a potential biomarker for EGFR-TKI sensitivity predictors, and implying that maintaining PHLPP expression during EGFR-TKI therapy may be a valuable strategy for delaying or overcoming drug resistance.

## Data Availability Statement

The raw data supporting the conclusions of this article will be made available by the authors, without undue reservation.

## Ethics Statement

The studies involving human participants were reviewed and approved by the Institutional Medical Ethics Review Board, Taizhou Hospital of Zhejiang Province. The patients/participants provided their written informed consent to participate in this study. The animal study was reviewed and approved by Institutional Animal Care and Use Committee, Taizhou Hospital of Zhejiang Province.

## Author Contributions

WW designed and performed most experiment, analyzed data, and wrote the manuscript, XX, KC, MC, and YM performed some experiments *in vitro* assay and *in vivo* assay. DL performed experiment on clinical samples. HY designed and provided leadership for the project and wrote the manuscript. All authors contributed to the article and approved the submitted version.

## Funding

This work was supported by Medical Science and Technology Project of Zhejiang Province, China (no. 2018KY887) and National Natural Science Foundation of China (no. 81874221). The funder had no role in the study design, the data collection and analysis, the interpretation of the data.

## Conflict of Interest

The authors declare that the research was conducted in the absence of any commercial or financial relationships that could be construed as a potential conflict of interest.

## Abbreviations

NSCLC, non-small cell lung cancer; LUAD, lung adenocarcinoma; PHLPP, pleckstrin homology domain leucine-rich repeat protein phosphatase; EGFR, epidermal growth factor receptor; IHC, immunohistochemical; GEO, Gene Expression Omnibus; PFS, progression-free survival; TKIs, tyrosine kinase inhibitors; DFS, disease-free survival; ATCC, American Type Culture Collection; IC50, the inhibition concentration of 50% cell viability; GR, gefitinib-resistant.
